# Muscle size and muscle fat infiltration in patients with newly diagnosed prostate cancer: a case–control study

**DOI:** 10.3389/fonc.2026.1708133

**Published:** 2026-02-25

**Authors:** Chao Wang, Hui Wang, Yufei Yu, Xinming Yang, Yu Wang, Kui Zhang, Shuangquan Sun, Lingfeng Wu, Daocheng Fang, Hui Li, Yi Yang, Xiao Chen, Shumei Bi, Hui Wen

**Affiliations:** 1 Department of Urology, Songjiang Hospital Affiliated to Shanghai Jiaotong University School of Medicine, Shanghai, China; 2 Department of Radiology, Affiliated Hospital of Nanjing University of Chinese Medicine, Nanjing, China

**Keywords:** case-control, muscle fat infiltration, muscle size, prostate cancer, sarcopenia

## Abstract

**Aims:**

Cancer is a recognized risk factor for sarcopenia. While some studies suggest that androgen deprivation therapy (ADT) may contribute to muscle loss in patients with prostate cancer (PCa), it remains unclear whether alterations in muscle composition are already present at the initial diagnosis, before any treatment begins. This study aimed to evaluate computed tomography (CT)–based muscle size and muscle fat infiltration in patients with newly diagnosed prostate cancer.

**Methods:**

A total of 143 patients with newly diagnosed PCa (January 2023–June 2024) were enrolled and matched 1:2 with 286 controls. CT attenuation and cross-sectional area of the erector spinae (ES) and full-layer muscle at the T12 level were measured. The muscle-to-spleen attenuation ratio (M/S) was calculated. Muscle fat infiltration was assessed based on muscle CT attenuation. Multivariable logistic regression analyses were performed to examine the association between PCa and the risk of low muscle CT attenuation or low muscle area.

**Results:**

In PCa patients, muscle CT attenuation, M/S ratio, ES area, and full-layer muscle area decreased with advancing age. Overall, muscle CT attenuation and M/S ratio were significantly lower in the PCa group than in controls. Both ES area and full-layer muscle area were also significantly reduced in PCa patients younger than 70years. A similar difference in ES area was observed in the 70–80-year age group. After full adjustment, PCa was significantly associated with an increased risk of low ES CT attenuation (OR=2.59, 95%CI: 1.25–5.39), low full-layer muscle CT attenuation (OR=7.14, 95%CI: 3.30–15.45), low full-layer muscle M/S ratio (OR=4.29, 95%CI: 2.01–8.87), and low ES area (OR=2.94, 95%CI: 1.33–6.53). Similar associations were observed in the non-diabetic subgroup.

**Conclusion:**

Newly diagnosed prostate cancer is associated with an increased risk of CT-based skeletal muscle fat infiltration and reduced muscle area.

## Introduction

Sarcopenia is a multifactorial syndrome characterized by progressive loss of skeletal muscle mass, strength, and function (1). Although often age-related, it has been increasingly identified in various cancer populations (2). Sarcopenia is linked to poor tolerance of treatment, higher complication rates, and reduced survival in many cancer types (3, 4). Growing evidence also shows a significant association between sarcopenia and urinary tract disorders, including urologic cancers. In patients with uro-oncological diseases, sarcopenia has been independently associated with more aggressive tumors (5), increased treatment-related complications, and worse survival outcomes (6–8).

Prostate cancer (PCa) is the second most common malignancy in men worldwide (9, 10), accounting for over 1.4 million new cases annually. It originates predominantly from prostate gland epithelial cells and is driven by androgen receptor signaling. Although localized PCa has a 5-year survival rate exceeding 99% (11), advanced stages—particularly castration-resistant prostate cancer—remain incurable. Patients receiving androgen deprivation therapy (ADT) often experience significant muscle loss (12–14), which occurs independently of weight change and is associated with increased non-cancer mortality risk (15). Studies further indicate that sarcopenia is linked to worse perioperative and oncologic outcomes across various PCa treatments, including radical surgery, radiotherapy, and systemic therapies (16, 17). The prevalence of sarcopenia in PCa patients has also been documented (18). However, a critical unanswered question is whether such alterations are already present in initial, treatment-naïve patients.

Muscle fat infiltration (MFI) has attracted growing scientific interest in recent years. It has been associated with various adverse clinical outcomes, including coronary microvascular dysfunction (19) and all-cause mortality (20, 21). The role of MFI in cancer patients has also been reported (22). A limited number of studies further suggest that MFI is linked to decreased overall survival and increased treatment-related complications in patients with prostate cancer (PCa) (23, 24). However, it remains unclear whether the prevalence of MFI is higher in newly diagnosed PCa patients compared with the general population.

Considering the typically prolonged survival of prostate cancer (PCa) patients, the high prevalence of sarcopenia in the elderly, and its substantial clinical implications, investigating the status of sarcopenia in this population is warranted. We hypothesized that PCa patients may exhibit reduced muscle size and lower muscle density compared with a community-based population. Therefore, in this case–control study, we compared the prevalence of CT-based low muscle size and muscle fat infiltration between PCa patients and general population controls.

## Methods

### Participants

Data were collected from patients with clinically confirmed PCa at our institution between January 2023 and June 2025, with their CT images completely including the midline level of the T12 vertebra. The patients were newly diagnosed PCa, had not received any cancer-related treatments, and had no other concomitant diseases (such as stroke, heart failure, and rheumatic and immune diseases), which may have additional impacts on muscle mass. Controls were randomly selected from the general population and matched to the cases in a 1:2 ratio based on a age- and body mass index (BMI)-based sampling/frequency matching. Furthermore, the timing of the CT examination was also aligned with that of the prostate cancer group. Control subjects were individuals who underwent chest CT for physical examinations between 2023 and 2025, with no history of cancer, stroke, heart failure, or rheumatic/immune diseases. This study was approved by the Institutional Review Board of the Songjiang Hospital Affiliated to Shanghai Jiaotong University School of Medicine, and no written informed consent was required due to its retrospective design.

### Data collection

Clinically relevant information, including age, gender, height, weight, BMI, and history of diabetes mellitus (DM), was collected from the electronic medical record system. Laboratory measurements—including alanine aminotransferase (ALT), albumin, creatinine, blood glucose, high-density lipoprotein cholesterol (HDL-c), low-density lipoprotein cholesterol (LDL-c), triglycerides (TG), and total cholesterol (TC)—were obtained after an 8-h fast. CT images were retrieved from our institution’s picture archiving and communication system (PACS).

### CT parameters

Patient images were evaluated on PACS by two radiologists who were blinded to the clinical information. For this study, unenhanced chest or abdominal CT scans were used. The CT acquisition parameters were as follows: 120 kV, automatic mAs, 64 × 0.64 mm collimation, and a pitch of 0.8. Images were reconstructed with a slice thickness of 1–2 mm and an interval of 0.625 mm.

### Muscle assessment

**Figure 1:**
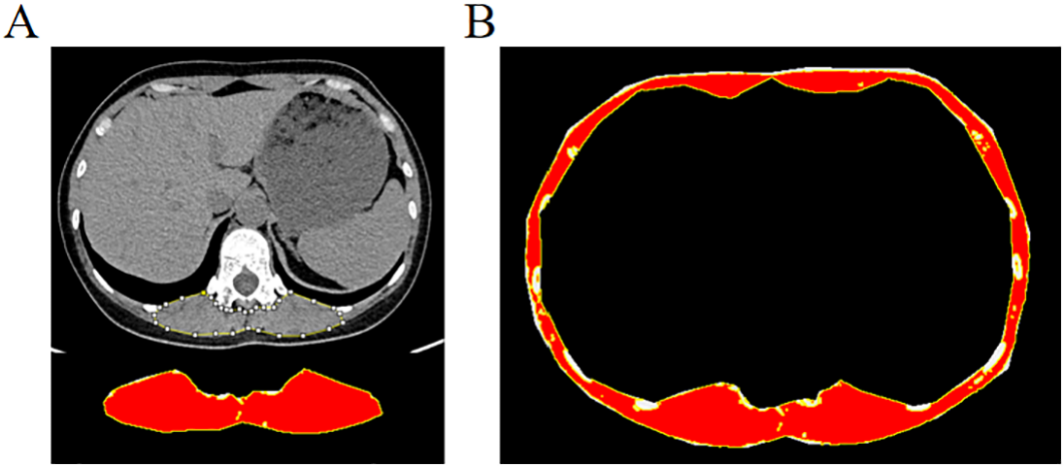
Illustrations of the measurements for muscle computed tomography attenuation. **(A)** Bilateral erector spinae; **(B)** Full-layer muscle.

On a single axial CT image at the midline level of the T12 vertebra, the CT attenuation and cross-sectional areas of the bilateral erector spinae (ES) and full-layer muscle (including the erector spinae, latissimus dorsi, external oblique, rectus abdominis, and intercostal muscles) were measured using ImageJ software. A threshold range of −29 to +150 HU was applied, consistent with previous literature (**Figure 1**) (25, 26). Muscle fat infiltration was defined as present when the CT attenuation of the ES was below 36 HU and that of the full-layer muscle was below 34 HU. These cutoff values were derived from the first quartile (Q1, 25th percentile) of the attenuation distribution within the entire study population due to the absence of universally established criteria. The quartile was calculated using the standard quartile function in SPSS and rounded to the nearest integer HU, resulting in operational thresholds of 36 HU for ES and 34 HU for full-layer muscle. Two radiologists independently performed blinded measurements on 30 randomly selected cases. Interobserver agreement, assessed by the intraclass correlation coefficient (ICC), was 0.995 for ES area and 0.996 for ES CT attenuation. For full-layer muscle, the ICC was 0.953 for area and 0.987 for CT attenuation.

Spleen CT attenuation was also measured, and the muscle-to-spleen CT attenuation ratio (M/S) was calculated. The cross-sectional muscle area was measured and normalized to stature (height^2^). Low muscle area was defined as an erector spinae (ES) area <30 cm² or <10.7 cm²/height², or a full-layer muscle area <72 cm² or <25.5 cm²/height². As there are no universally accepted criteria for defining reduced muscle area, these thresholds were determined based on the first quartile of the distribution within the entire study population.

### Statistical analysis

Qualitative data are presented as numbers, and continuous data as mean ± standard deviation (SD). Differences between groups were compared using independent-samples t-tests or the Mann-Whitney U test for continuous variables, and the chi-square test or Fisher’s exact test for categorical variables. Multivariable logistic regression was performed to assess the association between PCa and the risk of low muscle CT attenuation or low muscle area. In model2, adjustments were made sequentially for liver function, renal function, diabetes, and albumin (as a nutritional marker). Model3 further included low-density lipoprotein cholesterol, triglycerides, and high-density lipoprotein cholesterol. A sensitivity analysis was conducted after excluding participants with diabetes. A P-value <0.05 was considered statistically significant. All analyses were performed using SPSS version24.0.

## Results

### Characteristics of the participants

The characteristics of the study participants are presented in **Table 1**, and the distribution of muscle-related indices by quartile is shown in **Supplementary Table 1**. Low muscle density and low muscle area were defined based on the first quartile of the entire population. A total of 143 patients with PCa were included. Height and weight were lower in the PCa group than in the control group (*P*<0.01). Compared with controls, PCa patients had higher levels of creatinine and blood glucose and lower levels of albumin, ALT, HDL-c, TC, and LDL-c (all *P*<0.01). Muscle CT attenuation, muscle area, and the muscle-to-spleen attenuation (M/S) ratio—for both the erector spinae (ES) and full-layer muscle—were significantly lower in the PCa group than in the control group (all *P*<0.01). The proportions of participants with diabetes, ES CT attenuation<36HU, and full-layer muscle CT attenuation<34HU were all higher in the PCa group (all *P*<0.05). No significant differences were observed between the two groups in age, BMI, or TG (*P*>0.05). Among the PCa patients, 56 had a Gleason score≥8, 30 had a PSA level≥20ng/mL, 52 presented with lymph node metastasis, and 67 were classified as T3–T4 stage.

**Table 1:** Characteristics of the participants.

Variables	Prostate cancer (n = 143)	Control (n = 286)	p
Age (years)	74.03 ± 7.24	74.26 ± 8.81	0.79
Weight (kg)	67.86 ± 10.03	70.82 ± 9.43	0.004
Height (cm)	167.84 ± 5.50	169.56 ± 6.37	0.008
BMI (kg/m^2^)	24.08 ± 3.37	24.60 ± 2.70	0.09
ALT (U/L)	18.89 ± 9.65	21.24 ± 11.8	0.03
Albumin (g/L)	39.93± 3.92	43.49 ± 2.54	0.001
Creatinine (μmol/L)	85.46 ± 32.45	64.81± 21.43	< 0.001
Blood glucose (mmol/L)	6.01 ± 4.3	5.11 ± 1.84	< 0.001
HDL-c (mmol/L)	1.31 ± 0.52	1.54 ± 0.32	< 0.001
LDL-c (mmol/L)	2.40 ± 0.88	2.77 ± 0.77	< 0.001
TC (mmol/L)	4.22 ± 1.21	5.22 ± 1.05	< 0.001
TG (mmol/L)	1.66 ± 1.30	1.42 ± 1.01	0.06
Diabetes	21 (14.69%)	21 (7.34%)	0.02
ES CT attenuation (HU)	36.52 ± 6.87	43.07 ± 7.58	< 0.001
ES CT attenuation <36 HU	59 (41.2%)	51 (17.83%)	< 0.001
ES area (cm^2^)	33.18 ± 6.64	37.35 ± 8.13	< 0.001
Full layer muscle area (cm^2^)	77.86 ± 14.59	81.82 ± 12.83	0.004
Full-layer muscle CT attenuation (HU)	32.97 ± 5.73	40.12 ± 5.56	< 0.001
Full-layer muscle CT attenuation <34 HU	79 (55.2%)	37 (12.9%)	< 0.001
M/S ratio of ES	0.76 ± 0.16	0.80 ± 0.15	0.003
M/S ratio of full layer muscle	0.68 ± 0.14	0.75 ± 0.11	< 0.001
Gleason score (≥8)	56	/	
PSA		/	
<10 ng/ml	100	/	
10–20 ng/ml	13	/	
≥20 ng/ml	30	/	
Stage		/	
T1-T2	76	/	
T3-T4	67	/	
Lymph node metastasis	52		
High-risk	69	/	

ALT, alanine aminotransferase; BMI, body mass index; CKD, chronic kidney disease; ES, erector spinae; HDL-c, high-density lipoprotein cholesterol; LDL-c, low-density lipoprotein cholesterol; M/S, muscle/spleen; PSA, prostate-specific antigen; TC, total cholesterol.

**Figure 2:**
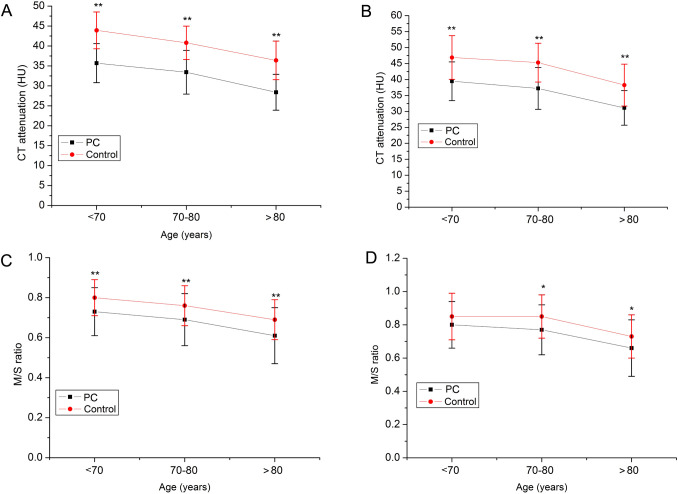
Muscle computed tomography attenuation between prostate cancer and control in different age groups. **(A)** ES CT attenuation. **(B)** All muscle CT attenuation. **(C)** M/S ratio of all muscle CT attenuation. **(D)** M/S ratio of ES CT attenuation. **p < 0.01; *p < 0.05.

**Figure 3:**
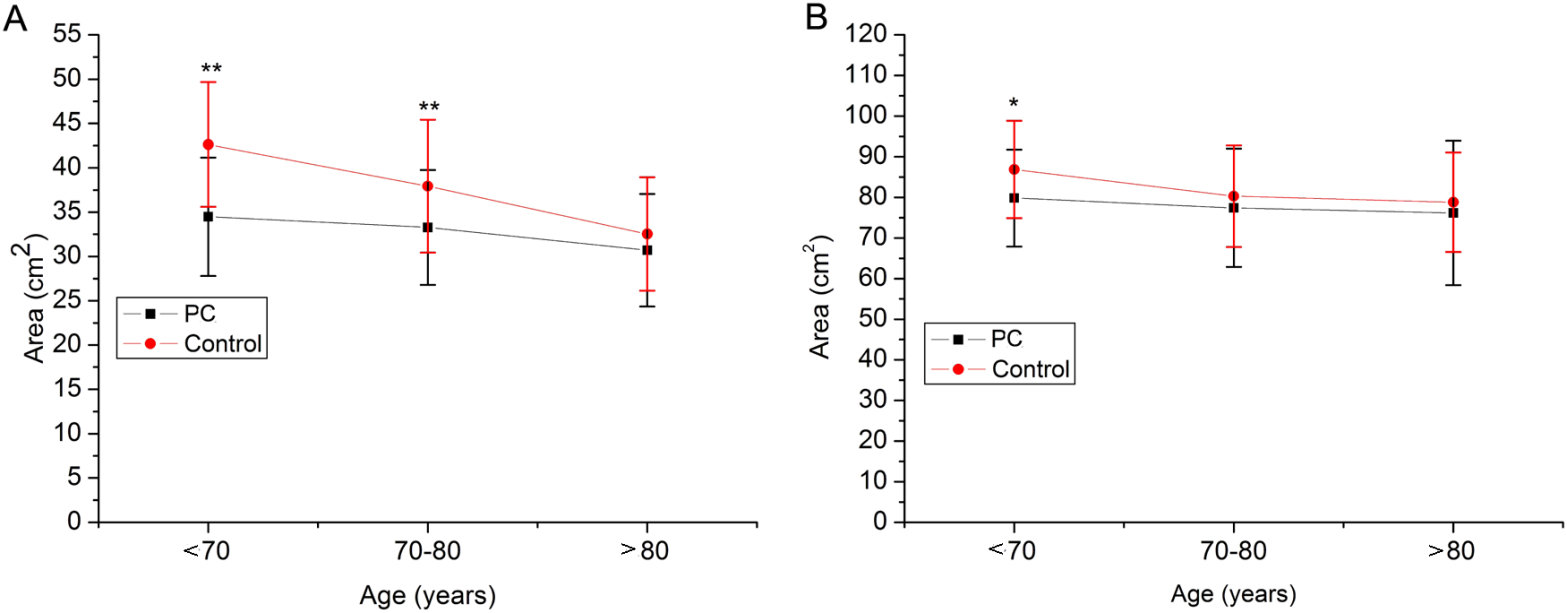
Muscle area between prostate cancer patients and control in different age groups. **(A)** ES muscle area. **(B)** All muscle area.

**Figure 2** displays the trends in muscle CT attenuation and the M/S ratio across age groups for both the PCa and control. A consistent age-related decline in muscle CT attenuation and the M/S ratio was observed in both groups. Moreover, the PCa group showed significantly lower values for both parameters than the control group in all age categories. With respect to muscle area, both the ES area and the full-layer muscle area were significantly lower in PCa patients aged <70years compared with controls (**Figure 3**). A similar reduction in ES area was also observed in the 70–80-year age group.

### The association between prostate cancer and risk of low muscle attenuation

Logistic regression analysis adjusted for age and BMI showed that PCa was associated with the risk of low ES CT attenuation (odds ratio [OR]=4.17, 95% confidence interval [CI]: 2.47–7.03), low full-layer muscle CT attenuation (OR=11.79, 95% CI: 6.74–20.64), low M/S ratio of ES (OR=2.14, 95% CI: 1.31–3.50), and low M/S ratio of full-layer muscle (OR=4.65, 95% CI: 2.77–7.79). After further adjustment for liver function, renal function, diabetes, and albumin, PCa remained significantly associated with the risk of low ES CT attenuation (OR=3.36, 95% CI: 1.76–6.41), low full-layer muscle CT attenuation (OR=8.85, 95% CI: 4.54–17.27), and low full-layer muscle M/S ratio (OR=3.68, 95% CI: 1.95–6.96). These associations persisted when additional potential confounders were included in the model (OR=2.59, 95% CI: 1.25–5.39; OR=7.14, 95% CI: 3.30–15.45; OR=4.29, 95% CI: 2.01–8.87, respectively) (**Table 2**). Sensitivity analysis restricted to the non-diabetic population yielded similar trends (**Table 3**), with PCa consistently associated with an increased risk of low ES CT attenuation, low full-layer muscle CT attenuation, and low full-layer muscle M/S ratio.

**Table 2:** Association between prostate cancer and the risk of low muscle attenuation.

Variables		Model 1	p	Model 2	p	Model 3	p
		OR (95%CI)		OR (95%CI)		OR (95%CI)	
ES CT attenuation < 36 HU	Age (years)	1.13 (1.09-1.18)	< 0.001	1.13 (1.09-1.18)	< 0.001	1.12 (1.08-1.17)	< 0.001
	BMI (kg/m^2^)	1.04 (0.96-1.12)	0.18	1.04 (0.96-1.12)	0.40	1.01 (0.93-1.10)	0.78
	PC (yes)	4.17 (2.47-7.03)	< 0.001	3.36 (1.76-6.41)	< 0.001	2.59 (1.25-5.39)	< 0.001
Full-layer muscle CT attenuation < 34 HU	Age (years)	1.11 (1.07-1.16)	< 0.001	1.11 (1.07-1.16)	< 0.001	1.12 (1.07-1.17)	< 0.001
	BMI (kg/m^2^)	1.09 (1.00-1.18)	0.06	1.09 (1.00-1.19)	0.05	1.06 (0.96-1.17)	0.26
	PC (yes)	11.79 (6.74-20.64)	< 0.001	8.85 (4.54-17.27)	< 0.001	7.14 (3.30-15.45)	< 0.001
M/S ratio of ES < 0.69	Age (years)	1.10 (1.06-1.14)	< 0.001	1.09 (1.06-1.14)	< 0.001	1.09 (1.05-1.13)	< 0.001
	BMI (kg/m^2^)	1.06 (0.98-1.14)	0.15	1.06 (0.98-1.14)	0.16	1.04 (0.95-1.13)	0.39
	PC (yes)	2.14 (1.31-3.50)	0.002	1.78 (0.96-3.31)	0.08	1.80 (0.89-3.67)	0.10
M/S ratio of full-layer muscle < 0.65	Age (years)	1.11 (1.07-1.15)	< 0.001	1.10 (1.06-1.14)	< 0.001	1.10 (1.06-1.147)	< 0.001
	BMI (kg/m^2^)	1.13 (1.04-1.23)	0.004	1.13 (1.04-1.19)	0.004	1.12 (1.03-1.23)	0.013
	PC (yes)	4.65 (2.77-7.79)	< 0.001	3.68 (1.95-6.96)	< 0.001	4.29 (2.01-8.87)	< 0.001

Model 2 was further adjusted for liver function (alanine aminotransferase), renal function (creatinine), diabetes, and albumin. Model 3 was further adjusted for low-density lipoprotein cholesterol, and high-density lipoprotein cholesterol.

CI, confidence interval; CT, computed tomography; ES, erector spinae; M/S, muscle/spleen; OR, odds ratio.

**Table 3:** Association between prostate cancer and the risk of low muscle attenuation in non-diabetes population.

Variables		Model 1	p	Model 2	p	Model 3	p
		OR (95%CI)		OR (95%CI)		OR (95%CI)	
ES CT attenuation < 36 HU	Age (years)	1.12 (1.08-1.16)	< 0.001	1.12 (1.07-1.16)	< 0.001	1.10 (1.06-1.15)	< 0.001
	BMI (kg/m^2^)	1.03 (0.95-1.12)	0.47	1.03 (0.95-1.12)	0.47	1.01 (0.92-1.10)	0.85
	PC (yes)	4.18 (2.42-7.21)	< 0.001	3.45 (1.73-6.90)	< 0.001	2.51 (1.11-5.64)	0.027
Full-layer muscle CT attenuation < 34 HU	Age (years)	1.11 (1.07-1.16)	< 0.001	1.10 (1.06-1.15)	< 0.001	1.10 (1.05-1.15)	< 0.001
	BMI (kg/m^2^)	1.07 (0.98-1.17)	0.14	1.07 (0.98-1.18)	0.13	1.05 (0.95-1.16)	0.26
	PC (yes)	11.97 (6.63-21.61)	< 0.001	8.80 (4.33-17.90)	< 0.001	5.90 (2.56-13.56)	< 0.001
M/S ratio of ES < 0.69	Age (years)	1.09 (1.05-1.13)	< 0.001	1.09 (1.05-1.13)	< 0.001	1.08 (1.04-1.12)	< 0.001
	BMI (kg/m^2^)	1.07 (0.99-1.16)	0.10	1.07 (0.99-1.16)	0.17	1.05 (0.97-1.15)	0.23
	PC (yes)	2.51 (1.22-3.49)	0.007	1.61 (0.82-3.16)	0.09	1.69 (0.77-3.71)	0.19
M/S ratio of full-layer muscle < 0.65	Age (years)	1.11 (1.07-1.15)	< 0.001	1.10 (1.06-1.14)	< 0.001	1.09 (1.05-1.14)	< 0.001
	BMI (kg/m^2^)	1.15 (1.05-1.25)	0.003	1.15 (1.05-1.26)	0.003	1.15 (1.04-1.26)	0.006
	PC (yes)	3.95 (2.28-6.85)	< 0.001	3.07(1.54-6.12)	0.001	3.20 (1.43-7.16)	0.005

Model 2 was further adjusted for liver function (alanine aminotransferase), renal function (creatinine), diabetes, albumin. Model 3 was further adjusted for low-density lipoprotein cholesterol, and high-density lipoprotein cholesterol.

CI, confidence interval; CT, computed tomography; ES, erector spinae; M/S, muscle/spleen; OR, odds ratio

### The association between prostate cancer and risk of small muscle size

Subsequently, in the age- and BMI-adjusted logistic regression analysis (model 1), PCa was associated with the risk of low ES area (OR=2.91, 95% CI: 1.68–5.05). This association remained significant in multivariable analyses after further adjustment (model 2, OR=2.58, 95% CI: 1.30–5.10; model 3, OR=2.94, 95% CI: 1.33–6.53) (**Table 4**). However, no significant association was observed between PCa and low full-layer muscle area in any of the models (*P*>0.05). Similar results were obtained when muscle size was normalized to height squared (for ES area in model 3, OR=2.54, 95% CI: 1.21–5.36). Sensitivity analysis restricted to the non-diabetic population showed consistent trends (**Table 5**), indicating that PCa was associated with the risk of low ES area, but not with low full-layer muscle area.

**Table 4:** Association between prostate cancer and risk of low muscle area.

Variables		Model 1	p	Model 2	p	Model 3	p
		OR (95%CI)		OR (95%CI)		OR (95%CI)	
ES area < 30 cm^2^	Age (years)	1.16 (1.11-1.21)	< 0.001	1.16 (1.11-1.21)	< 0.001	1.16 (1.11-1.21)	< 0.001
	BMI (kg/m^2^)	0.84 (0.77-0.92)	< 0.001	0.84 (0.76-0.92)	<0.001	0.85 (0.77-0.94)	0.001
	PC (yes)	2.91 (1.68-5.05)	< 0.001	2.58 (1.30-5.10)	0.007	2.94 (1.33-6.53)	0.008
ES area index < 10.7 cm^2^/m^2^	Age (years)	1.13 (1.09-1.17)	< 0.001	1.13 (1.08-1.17)	< 0.001	1.12 (1.07-1.16)	< 0.001
	BMI (kg/m^2^)	/	/	/	/	/	/
	PC (yes)	2.65 (1.59-4.39)	< 0.001	2.45 (1.30-4.64)	0.006	2.54 (1.21-5.36)	0.014
Full-layer muscle area < 72 cm^2^	Age (years)	1.06 (1.03-1.09)	< 0.001	1.06 (1.02-1.09)	0.002	1.07 (1.03-1.11)	< 0.001
	BMI (kg/m^2^)	0.76 (0.69-0.83)	< 0.001	0.75 (0.69-0.83)	<0.001	0.73 (0.66-0.82)	< 0.001
	PC (yes)	1.51 (0.92-2.50)	0.11	1.44 (0.77-2.69)	0.26	1.33 (0.63-2.81)	0.46
Full-layer muscle area index < 25.5 cm^2^/m^2^	Age (years)	1.02 (0.99-1.04)		1.02 (0.99-1.05)	0.19	1.03 (1.00-1.06)	0.06
	BMI (kg/m^2^)	/	/	/	/	/	/
	PC (yes)	1.24 (0.78-1.97)	0.36	1.39 (0.77-2.50)	0.27	1.31 (0.65-2.64)	0.45

Model 2 was further adjusted for liver function, renal function, diabetes, and albumin. Model 3 was further adjusted for low-density lipoprotein cholesterol, triglyceride, and high-density lipoprotein cholesterol.

CI, confidence interval; ES, erector spinae; OR, odds ratio.

**Table 5:** Association between prostate cancer and risk of low muscle area in non-diabetes population.

Variables		Model 1	p	Model 2	p	Model 3	p
		OR (95%CI)		OR (95%CI)		OR (95%CI)	
ES area < 30 cm^2^	Age (years)	1.15 (1.11-1.20)	< 0.001	1.15 (1.10-1.20)	< 0.001	1.15 (1.09-1.21)	< 0.001
	BMI (kg/m^2^)	0.83 (0.75-0.91)	< 0.001	0.83 (0.75-0.92)	< 0.001	0.85 (0.76-0.94)	0.001
	PC (yes)	2.84 (1.59-5.07)	< 0.001	2.85 (1.35-6.03)	0.006	3.22 (1.32-7.82)	0.01
ES area index < 10.7 cm^2^/m^2^	Age (years)	1.12 (1.08-1.16)	< 0.001	1.12 (1.08-1.17)	< 0.001	1.11 (1.06-1.15)	< 0.001
	BMI (kg/m^2^)	/	/	/	/	/	/
	PC (yes)	2.38 (1.39-4.06)	0.001	2.38 (1.30-4.64)	0.015	2.33 (1.02-5.32)	0.046
Full-layer muscle area < 72 cm^2^	Age (years)	1.05 (1.02-1.09)	0.002	1.05 (1.02-1.09)	0.004	1.06 (1.02-1.10)	< 0.001
	BMI (kg/m^2^)	0.74 (0.67-0.82)	< 0.001	0.74 (0.66-0.82)	<0.001	0.72 (0.64-0.81)	< 0.001
	PC (yes)	1.31 (0.77-2.24)	0.32	1.56 (0.80-3.06)	0.19	1.63 (0.70-3.83)	0.26
Full-layer muscle area index < 25.5 cm^2^/m^2^	Age (years)	1.02 (0.99-1.05)	0.22	1.02 (0.99-1.05)	0.17	1.03 (1.00-1.06)	0.098
	BMI (kg/m^2^)	/	/	/	/	/	/
	PC (yes)	1.14 (0.69-1.87)	0.62	1.36 (0.73-2.54)	0.33	1.34 (0.63-2.88)	0.45

Model 2 was further adjusted for liver function, renal function, albumin. Model 3 was further adjusted for low-density lipoprotein cholesterol, triglyceride, and high-density lipoprotein cholesterol.

CI, confidence interval; ES, erector spinae; OR, odds ratio.

## Discussion

In this study, we investigated whether the prevalence of low muscle size and muscle fat infiltration is higher in patients with PCa than in the general population. Using CT-derived measures of muscle attenuation, M/S ratio, and muscle area as indicators of muscle fat infiltration and mass, we examined their association with PCa. Our findings demonstrate that the prevalence of CT-based low muscle size and muscle fat infiltration was significantly higher in the PCa group than in controls, with a particularly pronounced difference observed for muscle fat infiltration.

The European Society for Clinical Nutrition and Metabolism (ESPEN) recommends early nutritional risk screening for all cancer patients, combined with objective assessment of body composition and physical function (27). However, current nutritional assessments predominantly rely on measures such as body weight and BMI, which do not fully capture changes in body composition (28, 29). Muscle mass is closely linked to nutritional status; as a key factor influencing muscle growth and function, reduced muscle mass often reflects underlying nutritional impairment (30).

Sarcopenia is defined as a progressive loss of muscle mass and function (31). Multiple studies have shown that sarcopenia is an important risk factor for reduced survival in cancer patients (32–34). A previous review highlighted that sarcopenia may serve as a significant predictor of disease progression and poor prognosis in PCa (35). CT imaging has become a primary method for assessing muscle mass in patients with various conditions, including cancer (26). Our study found that muscle mass at the level of the 12th thoracic vertebra (T12) was significantly lower in the PCa group than in the control group. Measurements of the erector spinae and full-layer muscles at T12 have been validated to reflect whole-body muscle mass (36, 37). The reduced muscle size observed at this level in our cohort is consistent with earlier reports. In the age-stratified analysis, ES muscle size was smaller in PCa patients under 80years of age. For full-layer muscle area, however, a significant reduction was only observed in PCa patients younger than 70years. No significant differences were found between PCa patients and controls among participants older than 80years.

Regarding PCa, current sarcopenia research has primarily focused on muscle mass, with relatively few evaluating muscle density. Skeletal muscle radiodensity, measured in HU on CT scans, serves as a key indicator of muscle fat infiltration (38). Emerging evidence suggests that CT-based myosteatosis may be a stronger prognostic marker for survival in cancer patients than CT-based sarcopenia alone (39, 40). Several studies have demonstrated that CT-derived muscle density can effectively assess muscle status and predict outcomes in cancer populations (22, 41). For instance, a recent study reported that decreased muscle radiodensity in patients with myeloma was associated with tumor progression and reduced survival (42). Our findings similarly indicate that PCa is associated with an increased risk of low muscle CT density. Given that advanced age itself contributes to muscle degeneration (43, 44), we performed an age-stratified analysis and observed that muscle radiodensity was consistently lower in PCa patients than in controls across all age groups. This suggests that muscle radiodensity or muscle fat infiltration may reflect muscle alterations in PCa patients more sensitively than muscle size alone.

### Strength and limitations

The main strength of this study is that, to our knowledge, it represents the first investigation in China to compare the prevalence of computed tomography-based low muscle mass and muscle fat infiltration between patients with PCa and a matched general population sample. Several limitations of this study should be acknowledged. First, as a retrospective, single-center study with a relatively limited sample size of PCa patients, the findings may be susceptible to selection bias. Second, potential confounding factors, such as comorbidities specifically related to PCa, were not routinely assessed and may have influenced the results. Third, quantifying muscle fat infiltration based solely on CT attenuation values may not be sufficiently accurate. Magnetic resonance imaging (MRI) is considered as the gold standard for evaluating muscle fat. Future studies utilizing MRI as a tool for quantifying muscle fat are needed. Fourth, selecting the control group from a “health check-up chest CT” cohort is indeed not equivalent to completely random sampling from the general community population. This approach may introduce healthy screenee or volunteer bias, potentially affecting the representativeness of the control group in terms of health status, health awareness, and socioeconomic factors relative to the broader population. Finally, the associations between PCa clinical outcomes, such as prognosis and recurrence, and muscle size or fat infiltration were not examined in this study.

In conclusion, our study showed that skeletal muscle fat infiltration was higher and muscle area was lower in PCa compared with the general population. The high prevalence of muscle fat infiltration and sarcopenia may offer new insights for the clinical management PCa.

## Data Availability

The original contributions presented in the study are included in the article/**Supplementary Material**. Further inquiries can be directed to the corresponding authors.

## References

[1] Kirk B Cawthon PM Arai H Ávila-Funes JA Barazzoni R Bhasin S . The conceptual definition of sarcopenia: delphi consensus from the global leadership initiative in sarcopenia (GLIS). Age Ageing. (2024) 53:afae052. doi: 10.1093/ageing/afae052, PMID: 38520141 PMC10960072

[2] Zhang FM Wu HF Shi HP Yu Z Zhuang CL . Sarcopenia and Malignancies: epidemiology, clinical classification and implications. Ageing Res Rev. (2023) 91:102057. doi: 10.1016/j.arr.2023.102057, PMID: 37666432

[3] Williams GR Dunne RF Giri S Shachar SS Caan BJ . Sarcopenia in the older adult with cancer. J Clin Oncol. (2021) 39:2068–78. doi: 10.1200/JCO.21.00102, PMID: 34043430 PMC8260902

[4] Zhang FM Song CH Guo ZQ Yu Z Weng M Zhou FX . Sarcopenia prevalence in patients with cancer and association with adverse prognosis: A nationwide survey on common cancers. Nutrition. (2023) 114:112107. doi: 10.1016/j.nut.2023.112107, PMID: 37356170

[5] Furberg H Bradshaw PT Knezevic A Olsson L Petruzella S Stein E . Skeletal muscle and visceral adipose radiodensities are pre-surgical, non-invasive markers of aggressive kidney cancer. J Cachexia Sarcopenia Muscle. (2024) 15:726–34. doi: 10.1002/jcsm.13429, PMID: 38263932 PMC10995262

[6] Guo Z Gu C Gan S Li Y Xiang S Gong L . Sarcopenia as a predictor of postoperative outcomes after urologic oncology surgery: A systematic review and meta-analysis. Urol Oncol. (2020) 38:560–73. doi: 10.1016/j.urolonc.2020.02.014, PMID: 32268990

[7] Mayr R Fritsche HM Zeman F Reiffen M Siebertz L Niessen C . Sarcopenia predicts 90-day mortality and postoperative complications after radical cystectomy for bladder cancer. World J Urol. (2018) 36:1201–7. doi: 10.1007/s00345-018-2259-x, PMID: 29520591

[8] Su W Wu Y Liao S Zhang Z Zhang Y Ou W . A nomogram including sarcopenia for predicting progression-free survival in patients with localized papillary renal cell carcinoma: A retrospective cohort study. Ann Surg Oncol. (2024) 31:5815–26. doi: 10.1245/s10434-024-15666-2, PMID: 38954088

[9] Bray F Laversanne M Sung H Ferlay J Siegel RL Soerjomataram I . Global cancer statistics 2022: GLOBOCAN estimates of incidence and mortality worldwide for 36 cancers in 185 countries. CA Cancer J Clin. (2024) 74:229–63. doi: 10.3322/caac.21834, PMID: 38572751

[10] Dee EC Iyengar R Narayan A Feliciano EJG Wu JF Ho FDV . National cancer system characteristics and prostate cancer outcomes: an analysis of global data. Prostate. (2025) 85:947–53. doi: 10.1002/pros.24901, PMID: 40235173

[11] Alba PR Gao A Lee KM Anglin-Foote T Robison B Katsoulakis E . Ascertainment of veterans with metastatic prostate cancer in electronic health records: demonstrating the case for natural language processing. JCO Clin Cancer Inform. (2021) 5:1005–14. doi: 10.1200/CCI.21.00030, PMID: 34570630

[12] Blow TA Murthy A Grover R Schwitzer E Nanus DM Halpenny D . Profiling of skeletal muscle and adipose tissue depots in men with advanced prostate cancer receiving different forms of androgen deprivation therapy. Eur Urol Open Sci. (2023) 57:1–7. doi: 10.1016/j.euros.2023.09.004, PMID: 38020528 PMC10658404

[13] Buffoni M Dalla Volta A Valcamonico F Bergamini M Caramella I D’Apollo D . Total and regional changes in body composition in metastatic hormone-sensitive prostate cancer patients randomized to receive androgen deprivation + Enzalutamide zoledronic acid. The BONENZA study. Eur Urol Oncol. (2025) 8:782–91. doi: 10.1016/j.euo.2025.02.006, PMID: 40300921

[14] Smith MR Saad F Egerdie B Sieber PR Tammela TL Ke C . Sarcopenia during androgen-deprivation therapy for prostate cancer. J Clin Oncol. (2012) 30:3271–6. doi: 10.1200/JCO.2011.38.8850, PMID: 22649143 PMC3434987

[15] McDonald AM Swain TA Mayhew DL Cardan RA Baker CB Harris DM . CT measures of bone mineral density and muscle mass can be used to predict noncancer death in men with prostate cancer. Radiology. (2017) 282:475–83. doi: 10.1148/radiol.2016160626, PMID: 27598538

[16] Jahrreiss V Laukhtina E D’Andrea D Shariat SF . The prognostic value of sarcopenia in patients with prostate cancer: a systematic review. Curr Opin Urol. (2021) 31:315–23. doi: 10.1097/MOU.0000000000000885, PMID: 33965982

[17] Meyer HJ Wienke A Surov A . CT-defined low-skeletal muscle mass as a prognostic marker for survival in prostate cancer: A systematic review and meta-analysis. Urol Oncol. (2022) 40:103.e9–103.e16. doi: 10.1016/j.urolonc.2021.08.009, PMID: 34483042

[18] Kovač MB Pavlin T Čavka L Ribnikar D Spazzapan S Templeton AJ . The trajectory of sarcopenia following diagnosis of prostate cancer: A systematic review and meta-analysis. J Geriatr Oncol. (2023) 14:101594. doi: 10.1016/j.jgo.2023.101594, PMID: 37482497

[19] Souza ACDAH Troschel AS Marquardt JP Hadžić I Foldyna B Moura FA . Skeletal muscle adiposity, coronary microvascular dysfunction, and adverse cardiovascular outcomes. Eur Heart J. (2025) 46:1112–23. doi: 10.1093/eurheartj/ehae827, PMID: 39827905 PMC13376131

[20] Miljkovic I Kuipers AL Cauley JA Prasad T Lee CG Ensrud KE . Greater skeletal muscle fat infiltration is associated with higher all-cause and cardiovascular mortality in older men. J Gerontol A Biol Sci Med Sci. (2015) 70:1133–40. doi: 10.1093/gerona/glv027, PMID: 25838547 PMC4553718

[21] Nachit M Horsmans Y Summers RM Leclercq IA Pickhardt PJ . AI-based CT body composition identifies myosteatosis as key mortality predictor in asymptomatic adults. Radiology. (2023) 307:e222008. doi: 10.1148/radiol.222008, PMID: 37191484 PMC10315523

[22] Aleixo GFP Shachar SS Nyrop KA Muss HB Malpica L Williams GR . Myosteatosis and prognosis in cancer: Systematic review and meta-analysis. Crit Rev Oncol Hematol. (2020) 145:102839. doi: 10.1016/j.critrevonc.2019.102839, PMID: 31877534

[23] Sheean PM O’Connor P Joyce C Vasilopoulos V Badami A Stolley M . Clinical features and body composition in men with hormone-sensitive metastatic prostate cancer: A pilot study examining differences by race. Prostate Cancer. (2022) 2022:9242243. doi: 10.1155/2022/9242243, PMID: 35693376 PMC9184233

[24] Yamashita S Kawabata H Deguchi R Ueda Y Higuchi M Muraoka S . Myosteatosis as a novel predictor of urinary incontinence after robot-assisted radical prostatectomy. Int J Urol. (2022) 29:34–40. doi: 10.1111/iju.14704, PMID: 34535917

[25] Hemke R Buckless C Torriani M . Quantitative imaging of body composition. Semin Musculoskelet Radiol. (2020) 24:375–85. doi: 10.1055/s-0040-1708824, PMID: 32992366

[26] Chianca V Albano D Messina C Gitto S Ruffo G Guarino S . Sarcopenia: imaging assessment and clinical application. Abdom Radiol (NY). (2022) 247:3205–16. doi: 10.1007/s00261-021-03294-3, PMID: 34687326 PMC8536908

[27] Muscaritoli M Arends J Bachmann P Baracos V Barthelemy N Bertz H . ESPEN practical guideline: Clinical Nutrition in cancer. Clin Nutr. (2021) 40:2898–913. doi: 10.1016/j.clnu.2021.02.005, PMID: 33946039

[28] Prado CM Lieffers JR McCargar LJ Reiman T Sawyer MB Martin L . Prevalence and clinical implications of sarcopenic obesity in patients with solid tumours of the respiratory and gastrointestinal tracts: a population-based study. Lancet Oncol. (2008) 9:629–35. doi: 10.1016/S1470-2045(08)70153-0, PMID: 18539529

[29] Prado CM Siervo M Mire E Heymsfield SB Stephan BC Broyles S . A population-based approach to define body-composition phenotypes. Am J Clin Nutr. (2014) 99:1369–77. doi: 10.3945/ajcn.113.078576, PMID: 24760978

[30] McGlory C van Vliet S Stokes T Mittendorfer B Phillips SM . The impact of exercise and nutrition on the regulation of skeletal muscle mass. J Physiol. (2019) 597:1251–8. doi: 10.1113/JP275443, PMID: 30010196 PMC6395419

[31] Cruz-Jentoft AJ Sayer AA . Sarcopenia. Lancet. (2019) 393:2636–46. doi: 10.1016/S0140-6736(19)31138-9, PMID: 31171417

[32] Lin WL Nguyen TH Huang WT Guo HR Wu LM . Sarcopenia and survival in colorectal cancer without distant metastasis: a systematic review and meta-analysis. J Gastroenterol Hepatol. (2024) 39:2250–9. doi: 10.1111/jgh.16681, PMID: 38986533

[33] Dogan O Sahinli H Duzkopru Y Akdag T Kocanoglu A . Is sarcopenia effective on survival in patients with metastatic gastric cancer? World J Gastro Oncol. (2024) 16:1861–8. doi: 10.4251/wjgo.v16.i5.1861, PMID: 38764843 PMC11099424

[34] Lin WL Nguyen TH Lin CY Wu LM Huang WT Guo HR . Association between sarcopenia and survival in patients with gynecologic cancer: A systematic review and meta-analysis. Front Oncol. (2023) 12:1037796. doi: 10.3389/fonc.2022.1037796, PMID: 36936273 PMC10016260

[35] Zhao T Mao W Hu M Yu Q Peng X Ji J . Advances in sarcopenia and urologic disorders. Front Nutr. (2024) 11:1475977. doi: 10.3389/fnut.2024.1475977, PMID: 39568720 PMC11578050

[36] Cao J Zuo D Han T Liu H Liu W Zhang J . Correlation between bioelectrical impedance analysis and chest CT-measured erector spinae muscle area: A cross-sectional study. Front Endocrinol (Lausanne). (2022) 13:923200. doi: 10.3389/fendo.2022.923200, PMID: 35928896 PMC9343984

[37] Tan L Ji G Bao T Fu H Yang L Yang M . Diagnosing sarcopenia and myosteatosis based on chest computed tomography images in healthy Chinese adults. Insights Imaging. (2021) 12:163. doi: 10.1186/s13244-021-01106-2, PMID: 34743259 PMC8572237

[38] Heymsfield SB Wang Z Baumgartner RN Ross R . Human body composition: advances in models and methods. Annu Rev Nutr. (1997) 17:527–58. doi: 10.1146/annurev.nutr.17.1.527, PMID: 9240939

[39] Antoun S Lanoy E Iacovelli R Albiges-Sauvin L Loriot Y Merad-Taoufik M . Skeletal muscle density predicts prognosis in patients with metastatic renal cell carcinoma treated with targeted therapies. Cancer. (2013) 119:3377–84. doi: 10.1002/cncr.28218, PMID: 23801109

[40] Kroenke CH Prado CM Meyerhardt JA Weltzien EK Xiao J Cespedes Feliciano EM . Muscle radiodensity and mortality in patients with colorectal cancer. Cancer. (2018) 124:3008–15. doi: 10.1002/cncr.31405, PMID: 29797673 PMC6033621

[41] Yamashita S Iwahashi Y Miyai H Iguchi T Koike H Nishizawa S . Myosteatosis as a novel prognostic biomarker after radical cystectomy for bladder cancer. Sci Rep. (2020) 10:22146. doi: 10.1038/s41598-020-79340-9, PMID: 33335232 PMC7747702

[42] Diallo TD Blessing AIL Ihorst G Möller MD Jungmann PM Bamberg F . Myosteatosis in multiple myeloma: a key determinant of survival beyond sarcopenia. Skeletal Radiol. (2025) 54:275–85. doi: 10.1007/s00256-024-04735-y, PMID: 38940940 PMC11652573

[43] Deschenes MR . Effects of aging on muscle fibre type and size. Sports Med. (2004) 34:809–24. doi: 10.2165/00007256-200434120-00002, PMID: 15462613

[44] Larsson L Degens H Li M Salviati L Lee YI Thompson W . Sarcopenia: aging-related loss of muscle mass and function. Physiol Rev. (2019) 99:427–511. doi: 10.1152/physrev.00061.2017, PMID: 30427277 PMC6442923

