# Supplementary material for: Muscle size and muscle fat infiltration in patients with newly diagnosed prostate cancer: a case–control study
Source: Front Oncol. 2026 Feb 25;16:1708133. doi: 10.3389/fonc.2026.1708133 (PMC12975419; doi:10.3389/fonc.2026.1708133)
Supplement: Supplementary file 1 [file Table1.docx]

# Muscle size and muscle fat infiltration in patients with newly diagnosed prostate cancer: a case-control study

Table 1 Quartile of muscle-related indices

|  | Q1 | Q2 | Q3 |
| --- | --- | --- | --- |
| ES CT attenuation (HU) | 35.91 | 40.65 | 47.03 |
| ES area (cm^2^) | 30.13 | 35.01 | 41.32 |
| Full layer muscle area (cm^2^) | 72.07 | 80.39 | 89.13 |
| Full-layer muscle CT attenuation (HU) | 33.59 | 38.03 | 42.31 |
